# Medication Adherence and Coping Strategies in Patients with Rheumatoid Arthritis: A Cross-Sectional Study

**DOI:** 10.1155/2019/4709645

**Published:** 2019-03-04

**Authors:** Carolin Berner, Ludwig Erlacher, Karl H. Fenzl, Thomas E. Dorner

**Affiliations:** ^1^2nd Medical Division, Rheumatology, Kaiser Franz Josef Hospital, SMZ-Süd, Kundratstrasse 3, 1100 Vienna, Karl Landsteiner Society of Autoimmunology and Rheumatism, Austria; ^2^Institute of Social Medicine, Centre for Public Health, Medical University of Vienna, Kinderspitalgasse 15/1, 1090 Vienna, Austria

## Abstract

**Objectives:**

The aim of this study was to determine if strategies for coping with illnesses, demographic factors, and clinical factors were associated with medication adherence among patients with rheumatoid arthritis (RA).

**Methods:**

This cross-sectional study was conducted at a Viennese rheumatology outpatient clinic on RA patients. Medication adherence was assessed using the Medication Adherence Report Scale. Strategies for coping with illness were assessed using the Freiburg Questionnaire for Coping with Illness.

**Results:**

Half (N=63, 52.5%) of the 120 patients included in the study were considered completely medication adherent. Female sex (odds ratio [OR]: 4.57, 95% confidence interval [CI]: 1.14 – 18.42), older age (54-65 yr vs. <45 yr OR: 9.2, CI:2.0-40.70; >65 yr vs. <45 yr OR 6.93, CI:1,17 – 40.87), middle average income (middle average income vs. lowest income class OR= 0.06, CI= 0.01-0.43), and shorter disease duration (5-10 yr vs. >10 yr OR= 3.53, CI= 1.04-11.95; 1-4 yr vs. >10 yr OR=3.71, CI= 1.02-13.52) were associated with higher medication adherence. Levels of active coping (15.57 vs. 13.47, p=0.01) or diversion and self-encouragement (16.10 vs. 14.37, p=0.04) were significantly higher among adherent as opposed to less adherent participants. However, in multivariate regression models, coping strategies were not significantly associated with adherence.

**Conclusions:**

Age, sex, monthly net income, and disease duration were found to be associated with an increased risk for medication nonadherence among patients with RA. Coping strategies such as active coping, diversion, and self-encouragement were associated with adherence in univariate models, but not when adjusted for demographic and clinical factors.

## 1. Introduction

In chronic diseases medication adherence is poor. About 50% of all chronically ill patients do not take their medication as prescribed and consequences of nonadherence are far-reaching including compromised effectiveness of treatment and increase in healthcare costs [1]. The problem with medication adherence gets augmented with multiple morbidity, comorbidity, and polypharmacy [2]. Despite extensive evidence regarding drug efficacy and the risk of long-term harm from uncontrolled disease progress, also in the rheumatoid arthritis (RA) population poor medication adherence has been reported to be a major issue, with only 30 to 80% of patients taking their medication as prescribed [3]. Societal impact due to medication nonadherence in RA implies losses in productivity due to illness recurrence and increased healthcare costs [4, 5]. Also adherent patients have more favourable outcomes [6], including better disease control, higher remission rates, improved physical function [7, 8], slower disease progression, and lower risk to therapy escalation to more aggressive treatment [9, 10].

Poor medication adherence is multifactorial and multiple causes are to be considered. Besides demographic and clinical factors, the contribution of psychological factors has been taken into account [10–13]. Risk factors are described inconsistently across literature. Demographic variables such as age, ethnicity, and marital status have been shown to influence medication adherence while other studies have refuted these findings. Also clinical and psychological factors have been suggested to influence the degree of adherence, including functional ability, individual pain rating, duration of disease, level of self-efficacy, degree of social support, illness beliefs, and beliefs about medication, adverse medication effects, and coping strategies [10, 14–16].

Coping strategies are important tools to manage disease flares of any kind. Because an individual's coping approach can impact how he or she cognitively appraises a situation or event and ultimately derives stress from it, coping skills could play an integral role in adherence behavior [17].

How well chronically ill patients adhere to their medication regime and certain coping strategies has been suggested to be influenced by coping strategies. For example, denial was commonly used by asthma patients with poor medication adherence [18]. Also, coping style seems to be an individual difference that distinguishes adherent from nonadherent behaviors in patients with schizophrenia [19].

However, little is known about the most effective type of coping and its association with medication adherence in chronic disease. To our knowledge only few studies included coping strategies to evaluate predictive factors of medication adherence in RA patients [10, 20], raising the need to further explore the association of illness coping strategies and medication adherence in RA patients.

The primary aim of this cross-sectional study was to determine if coping strategies together with demographic factors and clinical factors were associated with medical adherence in RA.

## 2. Patients and Methods

### 2.1. Design and Patients

This cross-sectional study was conducted in the Rheumatology Department at the Kaiser Franz Josef Hospital in Vienna, Austria. Between October 2013 and January 2014, RA patients were consecutively screened by their rheumatologist for eligibility during regular outpatient visits. Patients were eligible to participate if they had a confirmed diagnosis of RA according to ACR/EULAR classification criteria (2010) and a stable disease course with an RA-drug regimen for at least three months prior to study entry. Participants had to be ≥18 years old, had to live at home independently, and had to be able to read and understand the questionnaires. Patients were excluded if they had any significant comorbidity (e.g., advanced cancer, serious mental health problems).

### 2.2. Measures

#### 2.2.1. Sociodemographic and Clinical Characteristics

Sociodemographic factors measured were age, sex, marital status, educational level, and monthly net income. Clinical factors measured were disease duration, RA treatment, and number of other medications taken.

#### 2.2.2. Self-Reported Medication Adherence

Participants' adherence to their prescribed RA medication was assessed using the Medication Adherence Report Scale (MARS-5) which is a short version of the MARS [21, 22] and has been validated and widely used in a variety of diseases (e.g., Stroke, DM II, hypertension) [23–25]. This questionnaire measures self-reported adherence to medication and was translated into German and validated in German [22]. The scale comprises 5 items formulated as questions including the following: “I forget to take my medication”, “I change dosages”, “I stop taking my medicine for a while”, “I skip a dosage”, and “I take less drugs than prescribed”. Each item is rated on a 5-point Likert-type scale with 5 = never, 4 = rarely, 3 = sometimes, 2 = often, and 1 = very often. The MARS overall score was obtained by summing all responses to one score. The score ranges from 5 to 25 with higher scores indicating more adherent behavior. No definite cut-off point to define adherent versus nonadherent medication has been provided by the scale developers and it varies across studies [26]. In this study participants who ticked off “never” in all items, resulting in a MARS score of 25, were classified as adherent, and all other patients were classified as not completely adherent. This cut-off point corresponds to a dichotomization at the median. In the present study, the MARS proved acceptable internal consistency with Cronbach's alpha = 0.76.

### 2.3. Coping

For assessing the participants' coping strategies the Freiburg Questionnaire of Coping with Illness (FQCI) was used. The FQCI is one of the most commonly used methods for the assessment of coping in the German speaking countries. This self- assessment questionnaire has been validated with large samples of patients suffering from chronic diseases including arthritis and chronic back pain [27, 28]. It assesses coping strategies on the cognition, emotional, and behavioral level. The questionnaire comprises 35 items which are rated on a 5-point Likert scale ( 1 = does not apply at all, 5 = applies very much) and allocated to the following 5 dimensions of coping: (1) ‘depressive coping', (2) ‘active problem orientated coping', (3) ‘distracting and self-encouragement', (4) ‘consoling with religion and searching for the meaning of the illness', (5) ‘denying, dissimulating, and wishful thinking'. A mean value will be calculated for each dimension [29]. Crohnbach's alpha for the FQCI in this study ranged from 0.56 (religiousness and search for meaning) to 0.82 (depressive coping).

### 2.4. Statistical Analysis

All statistical analyses were performed using IBM SPSS Statistics, version 22.0. An a priori significance level of 0.05 was used to determine significance for all analyses. MARS scores were calculated for each participant and dichotomized to be adherent (MARS score=25) or not completely adherent (MARS score ≤24). Data were tested for suitability prior to parametric analysis. Characteristics and coping strategies of nonadherent and adherent participants were compared using independent samples t-tests, Wilcoxon rank sum test, or ANOVA analysis for continuous variables and Chi-square analyses for categorical variables with post hoc testing where appropriate. Variables that were identified to differ at < 0.2 level of significance and coping strategies were included in a binary logistic regression to identify independent risk factors for medication nonadherence.

### 2.5. Ethical Considerations

The study was conducted in accordance with the Helsinki Declaration. Ethical review committee approval was obtained from the City Government of Vienna (ERB no. EK-13-190-VK). Written informed consent was obtained from each patient before enrolment.

## 3. Results

### 3.1. Demographics and Clinical Characteristics

The descriptive statistics for demographic and clinical characteristics are presented in Table 1. Within 4 months of recruitment, 120 patients could be included, the majority of which were female. Participants had an average age of 54 years, more than half of the patients were between 45 and 65 years, and approximately equal parts were younger than 45 and older than 65 years. Most participants had at least secondary school graduation, were either currently employed or regularly retired, and had a monthly net income between 1001 and 2000 € (1121-2240$). Some participants were early retired and/or received disability pension, and most participants were married or were living in a stable relationship. There was an even distribution of disease duration for the categories 1-4 years, 4-10 years, and more than 10 years. As expected, when considering the inclusion criteria and requirements of stable disease and therapy, only a small number of participants had a disease duration of less than 1 year. At time of inclusion, most patients received synthetic disease modifying drugs (sDMARDs), approximately one-third received biological DMARDs (bDMARDs) or a combination therapy, and the majority of the participants had at least one additional medication, other than RA therapy.

### 3.2. Relationship between Demographics, Clinical Characteristics, Coping Strategies, and Medication Adherence

MARS summary scores ranged from 16 to 25 with the median at 25, the 25th at 23, and the 50th and 75th percentiles both at 25. Using the cut-off point for adherence at < 25, approximately half of the participants were considered adherent to medication and the other half were not completely adherent. In detail (Table 2), 33.3% of all participants declared lower adherence by forgetting to take their medication. Fewer patients intentionally either changed dosage (26.7%) or took fewer drugs than prescribed (20.8%). Only a small amount of participants stopped taking their RA medicine for a while (8.3%) or skipped a dose (11.7%).

The demographic and clinical characteristics of adherent and not completely adherent participants are presented in Table 1. The percentage of adherent participants did not differ by marital status, educational level, monthly net income, RA therapy, or number of other medications.

For sex, age, and disease duration, however, significant differences between adherent and not completely adherent participants were found. Post hoc analysis revealed that significantly more women and less men were adherent (adjusted residuals 2.5 and -2.5, respectively), and when comparing the 3 different groups of age the group below 45 years had significantly more participants with lower adherence and less adherent participants (adjusted residuals 3.1 and -3.1). Also, among participants with a disease duration > 10 years there were significantly less adherent participants (adjusted residuals 2.6 and -2.6).

Descriptive statistics for coping strategies and their relationship to medical adherence are listed in Table 3. There were significant differences between active coping as well as diversion and self-encouragement scores of adherent as compared with not completely adherent participants. There were no differences in scales for depressive coping, religiousness, and trivialization.

### 3.3. Factors Associated with Medication Adherence

A binary logistic regression model was used to calculate odds of factors associated with medication adherence (Table 4). Odds ratios of all variables included in the model (sex, age, marital status, monthly net income, disease duration, and number of other medications) were mutually adjusted. In total 44.2% of variance (R2) could be explained by the model. Female participants were more likely to be adherent than males. When using age < 45 years as a reference in the age category and disease duration > 10 years in the disease duration category, older patients and patients with shorter disease durations were more likely to be adherent. However, this was not true for participants <1 year of disease duration. Participants with a monthly net income of 2001-3000€ (2241-3360$) were shown less likely to be adherent as compared to the lowest net income category. Marital status and further medication intake did not significantly influence medication adherence. Also, none of the coping strategies reached statistical significance in the interaction with medication adherence.

## 4. Discussion

The present study examined the association between patient characteristics as well as coping behavior and medication adherence in patients suffering from RA. Lower medication adherence was largely due to forgetting medication intake and intentional change in medication doses. Female sex, older age, middle average income, and disease duration were found to be predictors for medication adherence. Some dimensions of coping strategies were associated with medication adherence in univariate analyses, but not in multivariate analyses.

Depending on the method and cut-off levels used, medication adherence for RA patients described in literature ranged broadly [8, 14, 15, 30, 31]. Unfortunately, many different methods were used across studies to assess medical adherence, and the lack of an established cut point for the Medication Adherence Report Scale (MARS), which was applied in this study, limits comparability with other studies. For the split between medication adherence and not complete adherence in this study, the sample median was used as cut-off point [32], classifying only half of the participants as perfectly medication adherent.

Female participants had a considerably higher chance of being medication adherent. According to a literature review [3] the majority of studies did not report an association between sex and medication adherence in RA. Only one study [33] showed a negative association, and one longitudinal study [8] supports our finding. Due to relatively large confidence intervals (CI), no reliable conclusion can be drawn from our finding.

We found that older participants were more likely to be medication adherent. As hypothesized before [34], a reason for this finding might presumably be a busier lifestyle of younger participants with more focus on professional and social life than on their illness. It is however well known that age seems to affect adherence in different directions. Also earlier studies reported that older age is associated with higher rates of medication adherence in patients with RA [8, 34]. Other investigators in turn have shown contradictory results, with younger patients being more successful in terms of medication adherence [3]. It must be mentioned that our results on the association of age and medication adherence are limited due to uncertainty shown by large confidence intervals with the lower value close to 1, so that a genuine interpretation is difficult. In older patients, medication adherence may be impacted by multiple confounders such as comorbidity, polypharmacy, with a higher incidence of side effects, and not least neuropsychological impairment [35].

Although there was no significant difference in adherence when comparing the influence of monthly net income, the regression model showed a bigger chance of adherence for the participants with a net income of 2001-3000 € (2241-3360 $) when compared to the lowest income group. This effect could not be seen in the highest income group. Several previous studies included the general socioeconomic status in their analysis but did not find any associations with medication adherence [10, 36, 37]. One study found that adherent patients had more financial resources than nonadherent patients [16]. This factor however is presumably highly dependent and thus only interpretable with knowledge of the particular underlying healthcare system and whether medication supply depends on the patient's own financial resources or not.

Disease duration was the only disease related factor which had an impact on medical adherence. In the group of participants with disease duration over 10 years, significantly more participants reported nonadherence than adherence. For participants with disease duration less than 10 years, the risk of being nonadherent was considerably lower. To our knowledge at this current time only few studies [31, 38–40] have investigated the influence of disease duration on medical adherence and none of these studies showed any positive associations.

More than 200 variables have been identified by researchers to influence medication adherence but no consistent risk profile for nonadherence could be created over the time [3].

The drivers of nonadherence are complex but have been suggested to base on beliefs about a patients illness and medical treatment [41, 42]. Illness perception influences coping and self-management strategies in response to perception of a health threat [10]. This study is one of the few examining coping strategies in association with medication adherence. Previous studies [10, 20] found that patients receiving social support were more likely to adhere to medication and avoidance is related to lower compliance. Findings for emotionally coping were contradictory and no effect could be found for problem focused coping.

In this study, the comparison between adherent and not completely adherent participants revealed that, in the adherent group, levels of active coping or diversion and self-encouragement were significantly higher. Adherent participants thus use more active coping strategies (i.e., they report to seek information about illness and therapy) to reduce negative outcomes (i.e., disease flares). Interestingly adherent participants also reported more diverting (i.e., distract with something pleasant) and self-encouraging (i.e., seeking success, indulge oneself) behavior. However, in the multivariate regression model a higher chance for adherence could actually be seen for participants with active coping and diverging or self-encouraging behavior but not significant. Reason for that could be that higher adherence by higher coping strategy competence could be mediated through the variables identified as being clearly associated with adherence in our multivariate model such as sex, age, and disease duration.

This study was limited due to its cross-sectional and monocentric character. The study population primarily consisted of female participants, underrepresenting male patients but reflecting the characteristically population distribution of RA patient. Our study population features the advantage of a balanced distribution of age and disease duration >1 year. Even though a disease duration > 1 year is representative for most RA patients, medication adherence and the associated psychosocial factors are likely to be different in newly diagnosed as opposed to chronic RA patients.

The small sample size overall and in subgroups may not have provided sufficient statistical power to determine the influence of investigated variables on medication adherence, but only for the group of participants < 1 year of disease duration and monthly net income > 3000€ there were less than 10 events per variable. For all other variables, the rule of thumb that logistic models should be used with a minimum of 10 events per predictor variable applies [43]. The small sample size contributes to the relatively far confidence intervals of significant results in the logistic regression analysis.

The use of MARS without a predefined cut-off point to discriminate adherence from nonadherence and using the median may overestimate lower adherence. However, in our population distribution of MARS data is strongly skewed. MARS median at 24 points is close to the mean, as well as the 75% percentile at 25 points and the 25% percentile at 23 points.

In general, when using a cut-off point and a dichotomization of a metric scale, results constitute a simplified outline of the studied characteristics of a population. However, this is to the detriment of a detailed description, and important information might get lost.

Furthermore, with regard to self-reported variables, there is always the concern that individuals will overreport their level of adherence because of their desire for social conformity.

To conclude, the demographic characteristics of age, sex, and monthly net income and the clinical parameter of disease duration were associated with increased risk of lower medication adherence. Notably, these findings are not consistent across studies. Coping strategies such as active coping, diversion, and self-encouragement were associated with adherence in univariate models, but not when adjusted for demographic and clinical factors. Considering the study limitations, future studies with a larger sample and potentially more objective measures for medication adherence should be conducted to verify our findings.

## Figures and Tables

**Table 1:** Participant characteristics and medication adherence.

	N (% of total) N=120 (100%)	Medication adherent (% within variable) N=63 (52.5%)	Not completely medication adherent (% within variable) N=57 (47.5%)	p-values
Sex				
Female	99 (82.5)	57 (57.6) *∗*	42 (42.4) *∗*	0.016
Male	21 (17.5)	6 (28.6) *∗*	15 (71.4) *∗*	
Age				
< 45 years	29 (24.2)	8 (27.6) *∗*	21(72.4) *∗*	0.008
45 – 65 years	65 (54.2)	39 (60.0)	26 (40.0)	
> 65 years	26 (21.7)	16 (61.5)	10 (38.5)	
Marital status				
Single	16(13.3)	5 (31.3)	11 (68.8)	0.101
Married	69 (57.5)	35 (50.7)	34 (49.3)	
Divorced/living separated	24 (20.0)	17 (70.8)	7 (29.7)	
Widowed	11 (9.2)	6 (54.5)	5 (45.5)	
Educational level				
Compulsory/lower secondary	31 (25.8)	16 (51.6)	15 (48.4)	0.785
Secondary school/apprenticeship	68 (56.7)	34 (50.0)	34 (50.0)	
Higher education	9 (7.5)	6 (66.7)	3 (33.3)	
Other	12 (10.0)	7 (58.3)	5 (41.7)	
Monthly net income				
< 1000€ (1120$)	23 (19.2)	14 (60.9)	9 (39.1)	0.168
1001–2000€ (1121 -2240$)	68 (56.7)	38 (55.9)	30 (44.1)	
2001–3000€ (2241-3360$)	20 (16.7)	6 (30.0)	14 (70.0)	
> 3000€ (>3361$)	9 (7.5)	5 (55.6)	4 (44.4)	
Disease duration				
>10years	35 (29.2)	12 (34.4) *∗*	23 (65.7) *∗*	0.048
5-10 years	40 (33.3)	25 (62.5)	15 (37.5)	
1-4 years	37 (30.8)	20 (54.1)	17 (45.9)	
<1year	8 (6.7)	6 (75.0)	2 (25.0)	
RA treatment				
sDMARDs	77 (64.2)	42 (54.5)	35 (45.5)	0.246
bDMARDs	8 (6.7)	2 (25)	6 (75)	
DMRAD + Biologic	30 (25)	15 (50)	15 (50)	
Corticosteroids	5 (4.2)	4 (80)	1 (20)	
Number of other medications				
No other medications	34 (28.3)	16 (47.1)	18 (52.9)	0.120
1–2 other medications	45 (37.5)	28 (62.2)	17 (29.8)	
3-4 other medications	19 (37.5)	6 (31.6)	13 (68.4)	
>4 other medications	22 (18.3)	13 (59.1)	9 (40.9)	

Descriptive statistic of demographic and clinical variable distribution, medication adherence, and their association with demographic and clinical variables. Values are absolute numbers (and percent of total or percent within variable).

*∗* means significantly different from expected value in post hoc testing.

sDMARDs: synthetic disease-modifying anti-rheumatic drugs; bDMARDs: biologic disease-modifying anti-rheumatic drugs.

**Table 2:** Medication adherence report (MARS).

	Never	Seldom	Sometimes	Often	Always
I forget to take my medication	80 (66.7)	33 (27.2)	5 (4.2)	2 (1.7)	0 (0)
I change dosages	88 (73.3)	21 (17.5)	10 (8.3)	1 (0.8)	0 (0)
I stop taking my medicine for a while	110 (91.7)	4 (3.3)	6 (5.0)	0 (0)	0 (0)
I skip a dosage	106 (88.3)	7 (5.8)	7 (5.8)	0 (0)	0 (0)
I take less drugs than prescribed	95 (79.2)	19 (15.8)	6 (5.0)	0 (0)	0 (0)

Descriptive statistics for medical adherence report (MARS) items. Values are absolute numbers (and percent of total number of participants).

**Table 3:** Coping strategies and medication adherence.

Coping strategies	Mean (SD) Min-Max	Adherent N=63	Not completely adherent N=75	p-values
Depressive coping	8.46 (3.9) 4-19	8.35 (3.8)	8.58 (4.1)	0.8
Active coping	14.59 (4.5) 5-25	15.57 (4.7)	13.47 (4.1)	0.01
Distracting and self-encouragement	15.27 (4.5) 5-25	16.10 (4.7)	14.37 (4.0)	0.04
Religion and meaning of illness	13.41(3.8) 5-24	13.68 (3.8)	13.11(3.8)	0.21
Denying, dissimulating, wishful thinking	6.79 (3.1) 3-15	6.98 (3.4)	6.58 (2.9)	0.69

Descriptive statistic of coping and association of coping strategies and medical adherence. Values are means (standard-deviation).

**Table 4:** Factors associated with medication adherence.

Factor	Odds ratio	95% Confidence interval (for the Odds/ExpB)
Sex		
Male	1	Referent
Female	4.57*∗*	1.14 – 18.42
Age categories		
<45 years	1	Referent
45-65 years	9.2*∗*	2.06 – 40.79
>65 years	6.93*∗*	1.17 – 40.87
Marital status		
Single	1	Referent
Married	1.45	0.31 – 6.85
Divorced/living separated	3.01	0.49 – 18.53
Widowed	2.26	0.27 – 18.83
Monthly net income		
< 1.000€ (1120$)	1	Referent
1.001–2000€ (1121 -2240$)	0.31	0.08 – 1.24
2001–3000€ (2241-3360$)	0.06*∗*	0.01 – 0.43
> 3000€ (>3361$)	0.49	0.05 – 4.84
Disease duration		
>10 years	1	Referent
5-10 years	3.53*∗*	1.04 – 11.95
1-4 years	3.71*∗*	1.02 – 13.52
<1 year	2.50	0.30 – 21.29
Number of other medications		
No other medications	1	Referent
1-2 other medications	1.78	0.56 – 5.66
3-4 other medications	0.22	0.37 – 1.27
>4 medications	1.55	0.34 – 7.22
Coping strategy (per point)		
Depressive coping	1.06	0.89 – 1.27
Active coping	1.06	0.91 – 1.24
Diversion and self-encouragement	1.15	0.96 – 1.38
Religiousness	0.87	0.73 – 1.05
Minimizing problems	0.86	0.71 – 1.05

Odds Ratios of medication nonadherence for all participants. Odds were calculated with binary logistic regression and all variables mutually adjusted for the others.

*∗* means statistically significant at *α*=0.05 level.

## Data Availability

The datasets used and/or analysed during the current study are available from the corresponding author on reasonable request.
